# Preparation of site-specifically fluorophore-labeled polyubiquitin chains for FRET studies of Cdc48 substrate processing

**DOI:** 10.1016/j.xpro.2023.102659

**Published:** 2023-10-26

**Authors:** Cameron Williams, Ken C. Dong, Connor Arkinson, Andreas Martin

**Affiliations:** 1 Biophysics Graduate Group, University of California, Berkeley, CA 94720, USA; 2 California Institute for Quantitative Biosciences, University of California at Berkeley, Berkeley, CA 94720, USA; 3 Department of Molecular and Cell Biology, University of California at Berkeley, Berkeley, CA 94720, USA; 4 Howard Hughes Medical Institute, University of California at Berkeley, Berkeley CA 94720

**Keywords:** Biophysics, Molecular Biology, Molecular/Chemical Probes, Protein Biochemistry

## Abstract

A critical step in the removal of polyubiquitinated proteins from macromolecular complexes and membranes for subsequent proteasomal degradation is the unfolding of an ubiquitin moiety by the cofactor Ufd1/Npl4 (UN) and its insertion into the Cdc48 ATPase for mechanical translocation. Here, we present a stepwise protocol for the assembly and purification of Lys48-linked ubiquitin chains that are fluorophore labeled at specific ubiquitin moieties and allow monitoring polyubiquitin engagement by the Cdc48-UN complex in a FRET-based assay.

For complete details on the use and execution of this protocol, please refer to Williams et al. (2023).^1^

## Highlights

• Linkage-specific enzymatic synthesis of ubiquitin chains
• Ubiquitin labeling with fluorescent dyes
• Positional attachment of fluorescent dyes to ubiquitin moieties in polyubiquitin chains
• FRET-based assay for ubiquitin unfolding by Ufd1/Npl4 and insertion into Cdc48

## Abstract (editor highlights)

Publisher’s note: Undertaking any experimental protocol requires adherence to local institutional guidelines for laboratory safety and ethics.

## Graphical abstract

**Figure:**
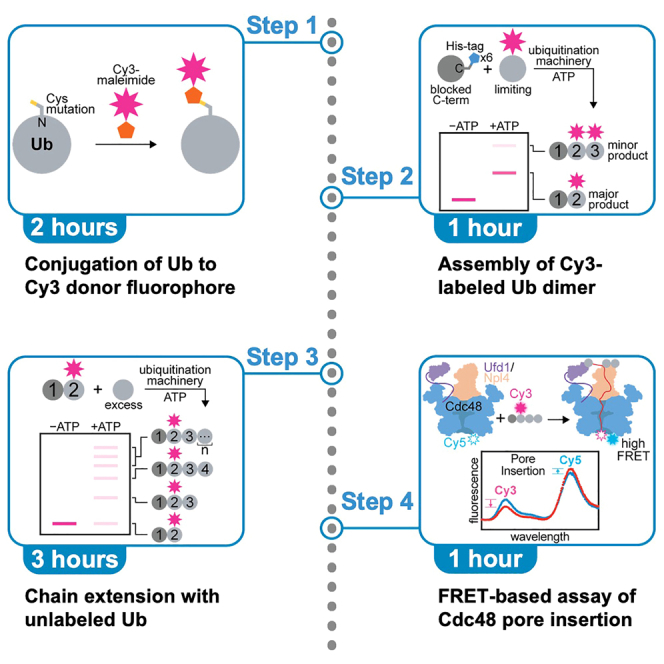

## Before you begin

The posttranslational modification of proteins with ubiquitin governs many vital processes in the cell, ranging from cell cycle control and DNA repair to protein homeostasis.^2^ Ubiquitin is assembled on proteins as a polymer called a polyubiquitin chain by the combined action of E1, E2, and E3 enzymes. Depending on the types of isopeptide linkages between individual ubiquitins, the chain can adopt different topologies.^3^ Since these topologies lead to different outcomes in the cell,^3^ methods to generate specific chains are necessary for probing the mechanisms of these different outcomes and pathways. Protocols are widely available for the *de novo* synthesis of free and substrate-attached polyubiquitin for *in vitro* studies.^4^ However, chains synthesized by these methods are often not well-defined and consist of identical moieties throughout their length. Mechanistic studies of proteins that bind polyubiquitin chain moieties in a positional or length-dependent manner could greatly benefit from a method for synthesizing chains with fluorophores or mutations at specific locations.

The protocol below describes the assembly of Lys-48-linked polyubiquitin chains containing a Cy3-donor fluorophore on the N-terminus of the most proximal ubiquitin (^Cy3^Ub-Ub_n_, written proximal-to-distal) or the first distally located ubiquitin (Ub-^Cy3^Ub-Ub_n_), however, any fluorophore dye can be attached using maleimide chemistry. We also describe how chain variants containing a substrate protein or a Met1-linked ubiquitin on the C-terminus of the most proximal ubiquitin are constructed. We used these specifically Cy3-labeled chains together with Cdc48-Ufd1/Npl4 (UN) complexes that had a Cy5 acceptor fluorophore conjugated at a position in the central processing channel of Cdc48 to monitor polyubiquitin-chain engagement in a FRET-based assay. Our studies revealed that Cdc48-UN unfolds and engages ubiquitins in a position- and linkage-specific manner.^1^

The first step of the protocol is to use site-directed mutagenesis for introducing a cysteine into ubiquitin, which is natively cysteine-free. Constructs with N-terminal cysteines used in this study are readily available, but cysteines for Cy3-labeling can also be introduced at other surface-exposed regions within ubiquitin. Two cysteine-containing ubiquitin constructs, one with and one without a C-terminal His_6_-tag, are required for the assembly of site-specifically labeled polyubiquitin chains. Polyubiquitin chains are then synthesized with a ubiquitination machinery in a one or two step manner for ^Cy3^Ub-Ub_n_ and Ub-^Cy3^Ub-Ub_n_, respectively. An example FRET-based assay with ^Cy5^Cdc48-UN is also presented at the end of this protocol, highlighting the power of positional labeling of ubiquitin chains.

### Expression and purification of tag-less wild-type ubiquitin and cysteine-containing ubiquitin

**Timing: 3 days**

1. Transform E. coli BL21 star (DE3) competent cells with the expression plasmids for tag-less wild-type S. cerevisiae ubiquitin or ubiquitin with a Met-Cys extension N-terminal to the native Met1 (MCM-Ub) by heat shock at 42°C in a water bath for 45 s. Recover cells for 1 h at 37°C in SOC medium.
2. Prepare 50 mL starter cultures in dYT (yeast extract, tryptone, and NaCl) with the appropriate antibiotic and grow 14 h at 37°C.
3. Inoculate 1 L of Terrific Broth containing kanamycin (50 μg/mL; kanamycin stock solution in water is stable for 12 months at 4°C or years at ‒20°C) with 8 mL of starter culture and grow at 37°C while shaking at 180 rpm until OD600 = 1.0.
4. Induce protein production for ∼18 h at 18°C with 1 mM IPTG (isopropylthio-β-galactoside, Gold Bio, Cat. No. I2481C; stock solution in water is stable at ‒20°C for several months).
5. Harvest cells by centrifugation at 3,500 rcf for 30 min at 18°C–20°C and resuspend the resulting pellets in NiA buffer (see materials and equipment section; NiA buffer can be stored for several weeks at 4°C) with added DNase I (Gold Bio, Cat. No. D-303-5, 50 units), lysozyme (RPI, Cat. No. L38100–25.0, 2 mg/mL), and protease inhibitors (1 μg/mL pepstatin (Gold Bio, Cat. No. P-020-100), 1 μg/mL aprotinin (Thermo Fisher Scientific, Cat. No. 78432) , 1 μg/mL leupeptin (Thermo Fisher Scientific, Cat. No. 78435), and 0.2 mg/mL AEBSF (4-(2-Aminoethyl)-benzenesulfonylfluoride hydrochloride, Gold Bio, Cat. No. A540-550). All enzyme and inhibitor solutions are stable for several months at ‒20°C. Inhibitor-containing NiA buffer should be stored at 4°C or on ice for a maximum of 1 day.
6. Lyse cells by sonication (15 s on, 45 s off, 2 min total on, 70% amplitude) and clarify the lysate by centrifugation at 27,000 rcf for 30 min at 4°C.
7. Add glacial acetic acid to the supernatant until a pH of 4.5 is reached, as monitored by pH paper.
8. Stir the solution for 30 min at 18°C–20°C to precipitate proteins other than ubiquitin.
9. Clarify the solution by centrifugation at 27,000 rcf for 30 min at 4°C.

***Note:*** Soluble ubiquitin will be found in the supernatant.

10. Dialyze the supernatant against 4 L of cation buffer A (see materials and equipment section; buffer can be stored for several weeks at 4°C) using a 3.5 kDa cutoff dialysis tubing (Spectra Por, Cat. No. 132725T). This step can be done for ∼ 14–16 h at 4°C.
11. Filter the dialyzed sample and load it onto a 5 mL HiTrap SP FF cation exchange chromatography column (Cytiva, Cat. No. 17115401) that has been equilibrated with cation buffer A. Elute ubiquitin from the column with a 0 mM–350 mM NaCl gradient over 20 column volumes using cation buffer A and cation buffer B on a Cytiva ÄKTA Pure FPLC or equivalent (see materials and equipment section for cation buffer B, which can be stored for several weeks at 4°C).

***Note:*** The first peak contains ubiquitin, while a second peak contains lysozyme from the lysis. Elution volumes for each peak can vary depending on how efficiently salt concentrations were reduced during the dialysis.

12. Combine and concentrate the fractions containing ubiquitin and further purify the protein by size-exclusion chromatography using a Superdex 75 16/600 column (Cytiva, Cat. No. 28989333) equilibrated in GF buffer (see materials and equipment section, GF buffer can be stored for several weeks at 4°C).

**CRITICAL:** TCEP at 1 mM was included in all purification steps above for MCM-Ub to keep the cysteine reduced for subsequent labeling with Cy3-maleimide.

### Expression and purification of wild-type ubiquitin and cysteine-containing ubiquitin with C-terminal his-tag

**Timing: 3 days**

13. C-terminally His_6_-tagged wild-type ubiquitin (Ub-His) and cysteine-ubiquitin (MCM-Ub-His) were expressed and cells lysed using the same steps 1–6 as above.
14. Following lysis, supernatants were incubated with Ni-NTA beads (Thermo Fisher Scientific, Cat. No. 88221) and batch bound for 30 min.
15. Beads were washed with NiA buffer, and proteins were eluted with NiA buffer plus 350 mM imidazole.
16. Eluates were concentrated with a 3 kDa Amicon spin filter (Millipore, Cat. No. UFC900396) and subjected to size-exclusion chromatography using a Superdex 75 16/600 column (Cytiva, Cat. No. 28989333) equilibrated in GF buffer.

**CRITICAL:** TCEP at 1 mM was included in all purification steps above for MCM-Ub-His to keep the cysteine reduced for subsequent labeling with Cy3-maleimide.

### Expression and purification of mouse E1 (Ube1), gp78RING-Ube2g2 E2-E3 chimera, and PreScission protease

**Timing: 3 days**

17. Ube1, the E2-E3 chimera gp78RING-Ube2g2, and PreScission protease were expressed and cells lysed using the same steps 1–6 as above for Ub and MCM-Ub.
18. Following lysis, supernatants were incubated with Ni-NTA beads and batch bound for 30 min.
19. Beads were washed with NiA buffer, and proteins were eluted with NiA buffer plus 350 mM imidazole.
20. The eluate for gp78RING-Ube2g2 was treated with TEV protease (at a TEV-to-E2-E3 w/w ratio of 1:200) for 14–16 h at 4°C to cleave the N-terminal His_6_-tag. The high-imidazole buffer was exchanged for NiA buffer by repeated concentration and dilution, and a subtractive step with Ni-NTA beads was used to remove uncleaved gp78RING-Ube2g2 and His-tagged TEV protease.
21. The flow-through containing gp78RING-Ube2g2 as well as the Ube1 and PreScission elutions from Ni-NTA beads were concentrated with spin filters and subjected to size-exclusion chromatography using a Superdex 75 16/600 (gp78RING-Ube2g2 and PreScission) or a Superdex 200 16/600 (Ube1) column equilibrated in GF buffer.

### Conjugation of Cy3 to cysteine-ubiquitin constructs

**Timing: 2–3 h**

22. MCM-Ub and MCM-Ub-His at 50 μM in GF buffer were incubated with 1 mM TCEP for 5 min to ensure cysteines are reduced for labeling.
23. Sulfo-Cy3-maleimide (20 mM stock in DMSO, Lumiprobe cat no: 21380) was added at a 10-fold molar excess (500 μM) and labeling occurred for 30 min at 18°C–20°C. See troubleshooting 1.
24. A 1 M stock of dithiothreitol (DTT) was added at 5 mM to quench unreacted maleimide dye, and the Cy3-labeled proteins were subjected to size-exclusion chromatography with a Superdex 75 increase 10/300 column equilibrated in GF buffer. Troubleshooting 2.
25. Labeling efficiency for ^Cy3^Ub and ^Cy3^Ub-His preparations was calculated by comparing the total protein concentration determined by a Bradford assay and the concentration of Cy3 dye determined by absorbance at the excitation wavelength maximum (555 nm). Troubleshooting 3.

**CRITICAL:** Ensure that purified protein stocks are free of thiol-containing contaminants that can react with maleimide. Quenching the labeling reaction with DTT is important, as unreacted Cy3-maleimide can damage chromatography columns.

## Key resources table

**Table:** 

REAGENT or RESOURCE	SOURCE	IDENTIFIER
**Bacterial and virus strains**		

Escherichia coli: BL21 Star (DE3) strain	QB3 MacroLab (UC Berkeley) or Thermo Fisher Scientific	N/A C601003

**Chemicals, peptides, and recombinant proteins**		

Sulfo-Cyanine3 maleimide	Lumiprobe	CAT# 21380
^Cy5^Cdc48	Williams et al.^1^	N/A

**Deposited data**		

Mendeley Data dataset: Uncropped SDS-PAGE gel images scanned for Cy3 fluorescence or Coomassie stain	This paper	https://data.mendeley.com/datasets/smzx3wz34w/draft?a=2969dbbb-3bd3-4f4a-a60b-3a0e5d0afba7

**Recombinant DNA**		

Plasmid: Ub (KAN)	Williams et al.^1^	pAM297
Plasmid: ^MCM^Ub (KAN)	Williams et al.^1^	pAM298
Plasmid: ^MCM^Ub^His^ (KAN)	Williams et al.^1^	pAM299
Plasmid: Ube1 (KAN)	Gift from Jorge Eduardo Azevedo	Addgene plasmid # 32534)
Plasmid: Cdc48^D602AzF^ (AMP)	Williams et al.^1^	pAM286
Plasmid: His_6_-TEV-gp78RING-Ube2g2 (KAN)	Blythe et al.^5^	pAM304
Plasmid: His-GST-PreScission (AMP)	Gift from QB3 MacroLab	pAM050

**Software and algorithms**		

Image Lab 6.1.0	Bio-Rad	N/A
Prism 9.3.1	GraphPad Software	N/A

## Materials and equipment

**Table:** NiA buffer

Reagent	Final concentration	Amount
1 M HEPES pH 7.6	60 mM	60 mL
2 M sodium chloride	200 mM	100 mL
2 M imidazole pH 7.6	20 mM	20 mL
ddH_2_O	N/A	820 mL
**Total**	**N/A**	**1 L**

**Table:** NiB buffer

Reagent	Final concentration	Amount
1 M HEPES pH 7.6	60 mM	60 mL
2 M sodium chloride	200 mM	100 mL
2 M imidazole pH 7.6	350 mM	175 mL
ddH_2_O	N/A	665 mL
**Total**	**N/A**	**1 L**

**Table:** Cation buffer A

Reagent	Final concentration	Amount
1 M sodium acetate pH 4.5	50 mM	50 mL
ddH_2_O	N/A	950 mL
**Total**	**N/A**	**1 L**

**Table:** Cation buffer B

Reagent	Final concentration	Amount
1 M sodium acetate pH 4.5	50 mM	50 mL
5 M sodium chloride	500 mM	100 mL
ddH_2_O	N/A	850 mL
**Total**	**N/A**	**1 L**

**Table:** GF buffer

Reagent	Final concentration	Amount
1 M HEPES pH 7.6	60 mM	60 mL
2 M sodium chloride	200 mM	100 mL
1 M magnesium chloride	10 mM	10 mL
50% glycerol	5%	100 mL
ddH_2_O	N/A	730 mL
**Total**	**N/A**	**1 L**

**Table:** Ubiquitination buffer

Reagent	Final concentration	Amount
1 M HEPES pH 7.4	20 mM	1 mL
1 M magnesium chloride	10 mM	0.5 mL
1 M DTT	0.5 mM DTT	25 μL
ddH_2_O	N/A	46 mL
**Total**	**N/A**	**50 mL**

**Table:** Assay buffer

Reagent	Final concentration	Amount
1 M HEPES pH 7.6	60 mM	3 mL
2 M sodium chloride	75 mM	1.875 mL
2 M potassium chloride	75 mM	1.875 mL
1 M magnesium chloride	10 mM	0.5 mL
ddH_2_O	N/A	42.75 mL
**Total**	**N/A**	**50 mL**

### Protein sequences

Ub: MQIFVKTLTGKTITLEVESSDTIDNVKSKIQDKEGIPPDQQRLIFAGKQLEDGRTLSDYNIQKSTLHLVLRLRGG.

^MCM^Ub:

MCMQIFVKTLTGKTITLEVESSDTIDNVKSKIQDKEGIPPDQQRLIFAGKQLEDGRTLSDYNIQKESTLHLVLRLRGG.

^MCM^Ub^His^:

MCMQIFVKTLTGKTITLEVESSDTIDNVKSKIQDKEGIPPDQQRLIFAGKQLEDGRTLSDYNIQKESTLHLVLRLRGGLEVLFQGPHHHHHH.

Cdc48^D602AzF^:

MGSSHHHHHHSQDPLEVLFQGPMGEEHKPLLDASGVDPREEDKTATAILRRKKKDNMLLVDDAINDDNSVIAINSNTMDKLELFRGDTVLVKGKKRKDTVLIVLIDDELEDGACRINRVVRNNLRIRLGDLVTIHPCPDIKYATRISVLPIADTIEGITGNLFDVFLKPYFVEAYRPVRKGDHFVVRGGMRQVEFKVVDVEPEEYAVVAQDTIIHWEGEPINREDEENNMNEVGYDDIGGCRKQMAQIREMVELPLRHPQLFKAIGIKPPRGVLMYGPPGTGKTLMARAVANETGAFFFLINGPEVMSKMAGESESNLRKAFEEAEKNAPAIIFIDEIDSIAPKRDKTNGEVERRVVSQLLTLMDGMKARSNVVVIAATNRPNSIDPALRRFGRFDREVDIGIPDATGRLEVLRIHTKNMKLADDVDLEALAAETHGYVGADIASLCSEAAMQQIREKMDLIDLDEDEIDAEVLDSLGVTMDNFRFALGNSNPSALRETVVESVNVTWDDVGGLDEIKEELKETVEYPVLHPDQYTKFGLSPSKGVLFYGPPGTGKTLLAKAVATEVSANFISVKGPELLSMWYGESESNIRDIFDKARAAAPTVVFLDELDSIAKARGGSLG∗AGGASDRVVNQLLTEMDGMNAKKNVFVIGATNRPDQIDPAILRPGRLDQLIYVPLPDENARLSILNAQLRKTPLEPGLELTAIAKATQGFSGADLLYIVQRAAKYAIKDSIEAHRQHEAEKEVKVEGEDVEMTDEGAKAEQEPEVDPVPYITKEHFAEAMKTAKRSVSDAELRRYEAYSQQMKASRGQFSNFNFNDAPLGTTATDNANSNNSAPSGAGAAFGSNAEEDDDLYSGSGSGSGSGSGLNDIFEAQKIEWHE.

His_6_-TEV-gp78RING-Ube2g2:

MGSSHHHHHHDYDIPTTENLYFQGHMEARFAVATPEELAVNNDDCAICWDSMQAARKLPCGHLFHNSCLRSWLEQDTSCPTCRMSLNIADNNRVREEGTGSHMAGTALKRLMAEYKQLTLNPPEGIVAGPMNEENFFEWEALIMGPEDTCFEFGVFPAILSFPLDYPLSPPKMRFTCEMFHPNIYPDGRVCISILHAPGDDPMGYESSAERWSPVQSVEKILLSVVSMLAEPNDESGANVDASKMWRDDREQFYKIAKQIVQKSLGL.

## Step-by-step method details

### Assembly and purification of polyubiquitin chains with a Cy3 fluorophore at the first ubiquitin

**Timing: 2–3 h**

**Figure 1:**
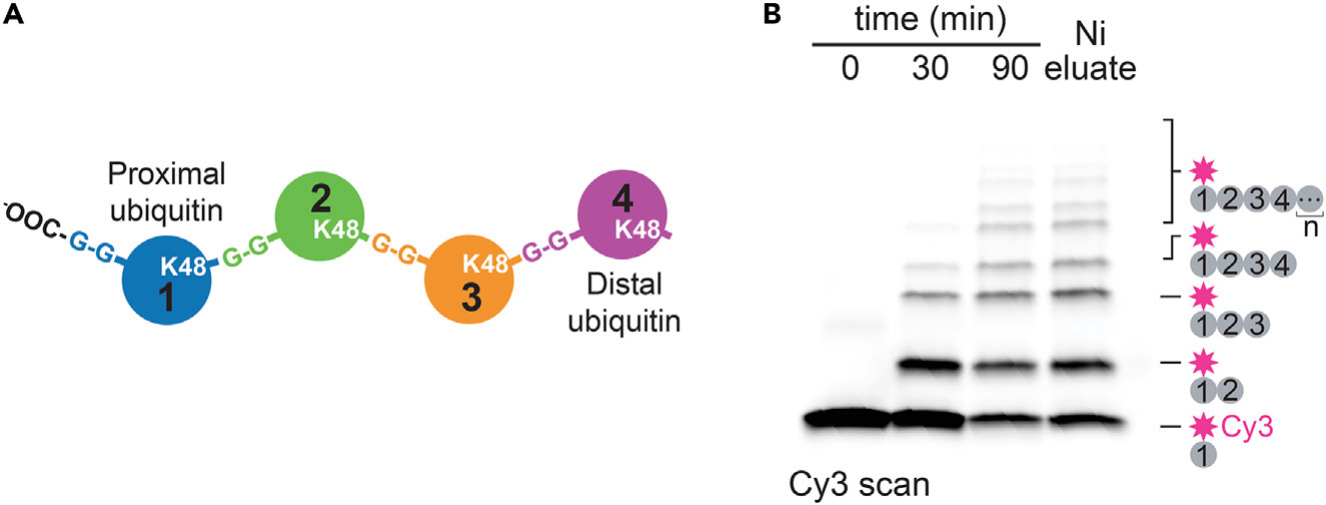
Synthesis and purification of polyubiquitin chains modified with Cy3 at the first ubiquitin (A) Schematic of a K48-linked tetra-ubiquitin chain, indicating the first, proximal ubiquitin with a free C-terminal glycine and the last, distal ubiquitin. (B) SDS-PAGE analysis of the assembly and purification of polyubiquitin chains conjugated to Cy3 (magenta star) through the first (proximal) ubiquitin. Additions of unlabeled ubiquitin to ^Cy3^Ub every 30 min led to a controlled elongation of the polyubiquitin chain. Gel samples were taken at the indicated time points of the ubiquitination reaction and following elution from Ni-NTA resin. Cy3-labeled chains were visualized by a scan for Cy3 fluorescence.

This major step describes the synthesis of Lys48-linked polyubiquitin chains with Cy3 conjugated to the most proximal ubiquitin in the chain (^Cy3^Ub-Ub_n_). The proximal ubiquitin is the ubiquitin closest to the substrate and in case of unanchored chains has a free C-terminal glycine, while the distal ubiquitin extends away from the substrate and represents the last moiety added to a ubiquitin chain (Figure 1A). For studies with Cdc48-UN, we aimed to generate chains that were heterogenous in length with about 2–6 ubiquitins. The C-terminal His-tag on ^Cy3^Ub-His serves two purposes. Since this construct does not contain a C-terminal Gly-Gly, Cy3-labeled ubiquitin can only serve as an acceptor for chain elongation and be present at the most proximal position in synthesized chains. The His-tag is also used to purify ^Cy3^Ub-Ub_n_ chains from untagged Ub_n_ chains and gp78RING-Ube2g2.

1. Create a mixture of 50 μM ^Cy3^Ub-His, 100 μM Ub, 1 μM mE1 and 20 μM of the E2-E3 chimera, gp78RING-Ube2g2, in ubiquitination buffer (see materials and equipment section).

***Note:*** Make sure to prepare ubiquitination buffer freshly each day and store at 4 °C to assure sufficiently reduced DTT.

2. Initiate chain elongation by adding ATP from a 500 mM ATP stock to a final concentration of 10 mM and react at 37°C for 30 min. This constitutes the first round of chain elongation. troubleshooting 4.
3. Add unlabeled ubiquitin to 100 μM and react at 37°C for another 30 min.
4. Repeat step 3 until the desired chain length has been reached as visualized on an SDS-PAGE gel.

**CRITICAL:** The E2-E3 chimera gp78RING-Ube2g2 is highly processive.^5^ Gradually adding unlabeled ubiquitin to ^Cy3^Ub-His ensures robust formation of chains with a tight length distribution (Figure 1B). The amount of unlabeled Ub added at each elongation step and the total number of additions might need to be optimized. Ideal reaction conditions will depend on the ubiquitin preparations and the method by which their concentrations were determined.

5. Dilute the reaction with NiA buffer and pass over Ni-NTA resin to selectively capture His-tagged ^Cy3^Ub-Ub_n_. Unlabeled Ub_n_ chains and gp78RING-Ube2g2 will remain in the flow-through.
6. Chains are eluted with the addition of NiB buffer.
7. NiB buffer is exchanged by repeated concentration and ten-fold dilution with GF buffer. This dilution step is done three times to ensure efficient buffer exchange.

### Assembly and purification of polyubiquitin chains with a Cy3 fluorophore at the second ubiquitin

**Timing: 3–4 h**

This major step describes the synthesis of Lys48-linked polyubiquitin chains with Cy3 conjugated to the ubiquitin positioned second in the chain, i.e., the first distal moiety that is directly attached via an isopeptide bond to the proximal ubiquitin (Ub-^Cy3^Ub-Ub_n_). For studies with Cdc48-Ufd1/Npl4, we aimed to generate heterogenous chains centered around Ub_5_ in length. The C-terminal His-tag on unlabeled MCM-Ub-His serves the same purpose as in the major step above. Only unlabeled Ub will be present at the most proximal position in a chain. Formation of Ub-^Cy3^Ub-Ub_n_, however, requires two steps. In this first step, limiting ^Cy3^Ub is added to MCM-Ub-His to form Ub-^Cy3^Ub and small amounts of Ub-^Cy3^Ub-^Cy3^Ub. After a Ni-NTA purification step, Ub-^Cy3^Ub is mixed with unlabeled Ub to form Ub-^Cy3^Ub-Ub_n_.

8. Create a mixture of 50 μM Ub-His, 5 μM ^Cy3^Ub, 1 μM E1 and 20 μM of the E2-E3 chimera, gp78RING-Ube2g2, in ubiquitination buffer.
9. Initiate elongation by adding ATP to 10 mM. Ideally, the major products will be Ub-^Cy3^Ub and unreacted Ub-His. troubleshooting 5.

**Figure 2:**
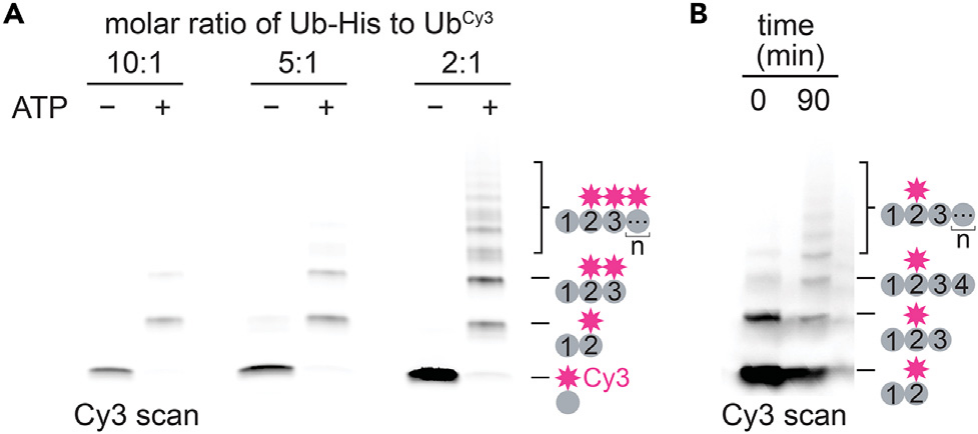
Synthesis and purification of polyubiquitin chains modified with Cy3 at the second ubiquitin (A) SDS-PAGE analysis of the assembly and purification of polyubiquitin chains conjugated to Cy3 (magenta star) through the second ubiquitin. Shown are the gel samples collected from ubiquitination reactions containing the indicated molar ratios of unlabeled Ub-His to Ub^Cy3^. Cy3-labeled chains were visualized by a scan for Cy3 florescence. (B) Elongation of Ub-^Cy3^Ub dimers with unlabeled ubiquitin. Shown are the gel samples at the indicated time points of the 90 min reaction with unlabeled ubiquitin added every 30 min. Cy3-labeled chains were visualized by a scan for Cy3 florescence.

**CRITICAL:** The goal of this part of the protocol is to add limiting amounts of ^Cy3^Ub to the ubiquitination reaction such that the major product is elongation of Ub-His by a single addition of Cy3-labeled ubiquitin (to form Ub-^Cy3^Ub). Conditions that lead to elongation of Ub-His by multiple additions of Cy3-labeled ubiquitin ^Cy3^Ub (forming Ub-^Cy3^Ub_n_) should be avoided. Small-scale test reactions can be performed using different molar ratios of Ub-His to ^Cy3^Ub to determine optimal ubiquitination reaction conditions (Figure 2A), which can vary depending on how the concentrations of ^Cy3^Ub and Ub-His samples were quantified.

10. Dilute the reaction with NiA buffer and pass over Ni-NTA resin to selectively capture Ub-^Cy3^Ub and unreacted Ub-His. Any ^Cy3^Ub_n_ and gp78RING-Ube2g2 will remain in the flow-through.
11. Use a 3 kDa MWCO spin filter to repeatedly concentrate and dilute into NiA buffer.
12. To synthesize unlabeled chains on the Ub-^Cy3^Ub dimer, set up a ubiquitination reaction in the same way as the one to form ^Cy3^Ub-Ub_n_ above.

***Note:*** Again, unlabeled Ub should be slowly added in batches over time (Figure 2B).

***Note:*** The amount of ubiquitin within each addition and the total number of additions may need to be optimized.

13. Pass the reaction over Ni-NTA to selectively capture Ub-^Cy3^Ub-Ub_n_ and elute chains with NiB buffer.
14. NiB buffer is exchanged for GF buffer by repeated concentration and dilution.

***Note:*** Contaminating unlabeled Ub-Ub_n_ will also be present following Ni-NTA elution.

### FRET-based assay of polyubiquitin engagement by Cdc48-Ufd1/Npl4

**Timing: 1 h**

This major section describes a FRET-based assay to monitor substrate engagement by Cdc48-UN that uses the Cy3-labeled polyubiquitin chains synthesized above. Initiation of substrate translocation by Cdc48-UN occurs through the ATP hydrolysis-independent insertion of the N-terminus of an unfolded ubiquitin moiety into the central pore of the Cdc48 ATPase.^6^ Cryo-EM structures of this initiation state revealed that the unfolded “initiator” ubiquitin was positioned proximal to two folded ubiquitin molecules bound to the Npl4 cofactor.^6^ Since no ubiquitins proximal to the engaged moiety were resolved in the structure, it was impossible to localize the initiator moiety within the length of the polyubiquitin chain, and we therefore sought to monitor pore insertion with a FRET-based assay for determining the positions of suitable initiator ubiquitins within a polyubiquitin chain. We synthesized Lys48-linked polyubiquitin chains labeled with a Cy3 donor fluorophore on the N-terminus of the first or second ubiquitin using the protocol described above and purified Cdc48 hexamers containing Cy5 acceptor fluorophores attached near the bottom of the central channel in the D2 ATPase domain, where the N-terminus of the initiator ubiquitin is located after initiation and prior to the onset of ATP-dependent translocation. Upon mixing of Cy3-donor-labeled chains with C5-acceptor-labeled Cdc48 in the presence of the UN cofactor and the non-hydrolyzable ATP analog ATPγS, we only observed a high-FRET state when the donor label was present at the second ubiquitin moiety, which indicated that Cdc48-UN preferentially engages ubiquitins distal in a chain, preceded by at least one other ubiquitin on the proximal side.^1^ This FRET-based assay not only revealed important kinetic and mechanistic details of a ubiquitous AAA+ ATPase motor, but also highlighted the power of methods for the assembly polyubiquitin chains with site-specific modifications.

15. Create a sample of 0.4 μM Cy3-labeled polyubiquitin chains (^Cy3^Ub-Ub_n_ or Ub-^Cy3^Ub-Ub_n_) in assay buffer (see materials and equipment section for assay buffer, which can be stored for several weeks at 4°C) supplemented with 1 mM ATPγS (ATPγS-containing assay buffer should be stored at 4°C and used within 1 day). This is the 2X polyubiquitin sample.
16. Create samples of 4 μM ^Cy5^Cdc48 (labeled through AzF incorporated at position 602 of *S. cerevisiae* Cdc48, see ref. 1 for detailed labeling protocol) with and without 4 μM UN in assay buffer supplemented with 1 mg/mL BSA and 1 mM ATPγS. These are the 2X ^Cy5^Cdc48 and ^Cy5^Cdc48-UN samples.
17. Create mixtures consisting of 6 μL of the 2X polyubiquitin sample and 6 μL of the 2X ^Cy5^Cdc48 or ^Cy5^Cdc48-UN sample. Incubate at 30°C in a thermocycler. The final concentrations are 0.2 μM ^Cy3^Ub-Ub_n_ or Ub-^Cy3^Ub-Ub_n_, 2 μM ^Cy5^Cdc48, 0 μM or 2 μM UN, and 0.5 mg/mL BSA.

***Note:*** Pore-insertion kinetics are rapid (∼3 s)^ref.^^1^ and therefore incubation of the mixture at 30°C for a few minutes is sufficient.

18. Transfer 10 μL of each mixture into wells of a 384-well black plate (Corning, Cat. No. CLS3820) pre-heated at 30°C and load into a microplate reader (BMG Labtech CLARIOstar Plus) equipped to measure fluorescence emission spectra of Cy3 and Cy5 following excitation at the absorbance maximum for Cy3.
19. Incubate at 30°C and scan wells for fluorescence emission between 525 nm and 725 nm using an excitation wavelength of 480 nm troubleshooting 6.

**CRITICAL:** Since both ^Cy3^Ub-Ub_n_ and Ub-^Cy3^Ub-Ub_n_ contain contaminating unlabeled polyubiquitin chains, pore-insertion experiments are performed with a 10-fold excess of ^Cy5^Cdc48-UN to avoid any competition between labeled and unlabeled chains. However, these conditions lead to technical challenges due to some extent of direct Cy5-acceptor excitation when exciting the Cy3 donor, which is further exacerbated by the fact that Cdc48 homohexamers can be modified with multiple Cy5 fluorophores depending on the labeling conditions and efficiency. If there is excessive direct excitation of Cy5, the Cy5 emission peak will dominate the spectrum and, due to spectral overlap, introduce some background fluorescence in the region of the Cy3 emission peak as well. To minimize Cy5 fluorescence emission resulting from direct excitation, experiments should be performed with an Cy3-donor excitation wavelength of 480 nm or lower.

**Figure 3:**
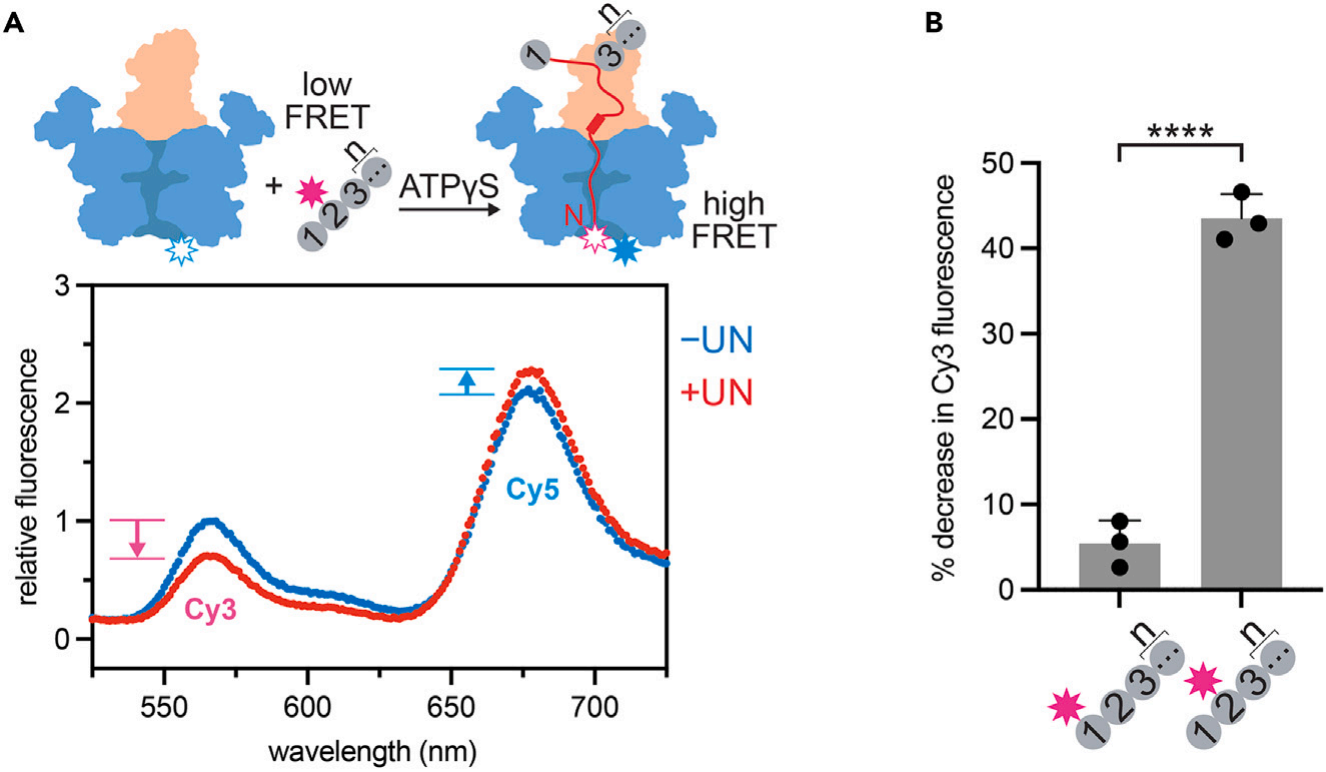
Example FRET-based assay for the initiator-ubiquitin insertion into the Cdc48-UN pore using site-specifically labeled polyubiquitin chains (A) Top: Schematic for the initiator-insertion assay, with Cdc48 shown in blue, the Ufd1/Npl4 cofactor in beige, the unanchored ubiquitin chain in gray, the Cy3 donor dye on ubiquitin as a magenta star, the unfolded initator ubiquitin in red, and the Cy5 acceptor dye at the bottom of the Cdc48 central channel as a blue star. Bottom: Fluorescence emission spectra after excitation at 480 nm for samples of Ub-^Cy3^Ub-Ub_n_ polyubiquitin chains and ^Cy5^Cdc48 with (red curve) and without UN (blue curve) in ATPγS. Fluorescence intensities were normalized relative to the Cy3-donor emission maximum at 568 nm for the spectrum in the absence of UN (-UN, blue dots), which was set to 1. Arrows indicate the direction of changes in Cy3- or Cy5-fluorescence intensity during the transition from a low-FRET to high-FRET state upon pore insertion of the initiator ubiquitin. (B) Pore insertion of a labeled ubiquitin located at the first or second position within polyubiquitin chain constructs. Bars represent the percent decrease in Cy3-donor fluorescence when UN is present in a sample of Cy3-labeled polyubiquitin chains (^Cy3^Ub-Ub_n_ or Ub-^Cy3^Ub-Ub_n_) and ^Cy5^Cdc48 in ATPγS. The individual values of percent decrease in fluorescence, their mean, and standard deviation are shown for N = 3 technical replicates. Statistical significance was calculated using an unpaired two-tailed Welch’s t test: ∗∗∗∗p < 0.0001.

20. Pore insertion under these conditions is best quantified by the quenching of donor fluorescence at the emission maximum observed in the presence of both ^Cy5^Cdc48 and UN (Figure 3):

$$\%\;decrease\;in\;donor\;emission=\frac{I_{D,-UN}-I_{D,+UN}}{I_{D,-UN}}\times 100$$

with I_D,-UN_ representing the fluorescence intensity of the Cy3 donor dye in the absence of Ufd1/Npl4, and I_D,+UN_ representing the fluorescence intensity of the Cy3 donor dye in the presence of Ufd1/Npl4. The Cy5 acceptor fluorescence is not a reliable indicator of FRET, as the observed changes are usually small due to direct excitation of excess Cy5 fluorophores. For a detailed description of the FRET analysis see Williams et al*.*^1^

## Expected outcomes

Methods for the assembly of polyubiquitin chains that possess specific properties in certain positions are of great value for *in vitro* mechanistic studies of biological systems involving ubiquitin. Herein, we report a protocol for the synthesis of Lys48-linked polyubiquitin chains that are modified with a fluorophore at the first or second moiety within the polymer. Our example FRET-based assay (Figure 3) illustrates how polyubiquitin chains site-specifically labeled with Cy3-donor fluorophores were crucial tools to dissect the mechanism of initiator-ubiquitin selection by Cdc48-UN.^1^ This method could be adapted for the synthesis of polyubiquitin chains containing site-specific mutations or modifications.

Polyubiquitin chains labeled with Cy3 at the first position (^Cy3^Ub-Ub_n_) are synthesized in a single step in which unlabeled ubiquitin is added progressively to Cy3-labeled ubiquitin with a C-terminal His-tag in the presence of ubiquitination machinery. The C-terminal His-tag on the fluorophore-labeled species is critical, because it restricts the labeled ubiquitin to the first position in the chain by preventing conjugation of this moiety to itself or to other ubiquitins during the course of the reaction. A tight distribution of polyubiquitin chain lengths is expected (Figure 1B), provided that unlabeled ubiquitin was added to the reaction mixture progressively over time. Cy3-labeled chains are then separated from unlabeled chains using a Ni-NTA affinity step. Yields of ∼ 50 μg of ^Cy3^Ub-Ub_n_ chains are achievable with this protocol.

Polyubiquitin chains labeled with Cy3 at the second position (Ub-^Cy3^Ub-Ub_n_) are synthesized in two steps. In the first step, a limiting amount of Cy3-labeled ubiquitin is added to unlabeled ubiquitin whose C-terminus is blocked with a His_6_-tag to yield a ubiquitin dimer with the labeled species in the second position (Ub-^Cy3^Ub). The molar ratio of these species should be optimized such that the single addition of Cy3-labeled ubiquitin to unlabeled ubiquitin is the major product (Figure 2A). In the second step, unlabeled ubiquitin is progressively added to a ubiquitination reaction containing the Cy3-labeled dimer until chains have reached the desired length (Figure 2B). Yields of ∼ 50 μg are achievable with this protocol with about one-tenth of the purified chains being Cy3-labeled.

Polyubiquitin chains site-specifically labeled with Cy3 donor fluorophores can be used to monitor the insertion of an initiator ubiquitin into Cdc48-UN in a FRET-based assay. *S. cerevisiae* Cdc48 is labeled with a Cy5 acceptor fluorophore at position 602, which is expected to be in close proximity to the Cy3 donor-labeled N-terminus of an initiator ubiquitin once it is inserted into the motor pore in the absence of ATP hydrolysis.^6^ Cy3-labeled polyubiquitin chains should be mixed with excess cofactor and Cy5-labeled Cdc48 to yield a robust decrease in Cy3 donor fluorescence due to energy transfer to the Cy5 acceptor (Figure 3A). Depending on the Cy5-labeling efficiency of Cdc48 and the extent of direct Cy5 excitation, an increase in the Cy5 acceptor fluorescence may also be observed (Figure 3A). Pore insertion is expected to only be observed for polyubiquitin chains labeled with Cy3 at the second position (Figure 3B), as the most proximal ubiquitin in a chain is not a suitable initiator.^1^

## Limitations

Our protocol is optimized for the assembly of Lys48-linked polyubiquitin chains that are site-specifically labeled with Cy3 donor fluorophores for FRET studies. This method could be extended to the synthesis of chains that are labeled with other fluorophores suitable for fluorescence polarization or single molecule measurements. Cysteine-maleimide chemistry was used to attach fluorophores in this protocol, but we have also successfully synthesized polyubiquitin chains from ubiquitins with fluorophores conjugated through the unnatural amino acid 4-azido-L-phenylalanine^7^ incorporated at different positions in the polypeptide. The synthesis of polyubiquitin chains containing point mutations or insertions at specific ubiquitins is also possible provided the ubiquitin mutant is compatible with the ubiquitination machinery. For example, we synthesized polyubiquitin chains in which Ile44 in the most proximal ubiquitin was mutated to Ala to disrupt binding to Ufd1.^1^ This method could also be extended to the assembly of chains with other linkage types (Lys11, Lys63, etc.) using linkage specific ligases.

Our biochemical synthesis results in a heterogeneous mixture of unlabeled and labeled polyubiquitin chains. Chemical approaches have also been employed to generate fluorescently labeled ubiquitin chains, including work pioneered by Huib Ovaa’s group (for a review, see Hameed et al.^8^), but these techniques require a lab capable of organic synthesis. In addition, recent work by Kathrin Lang’s group utilized evolved peptide ligases combined with incorporated unnatural amino acid to enable precise assembly of defined ubiquitin chains.^9^ While this is a powerful technique, usage of the sortase enzyme requires mutation of key amino acids at the C-terminus of ubiquitin shown to be critical for the survival in yeast.^10^ In addition these chains cannot be deubiquitinated, suggesting that the mutations at ubiquitin’s C-terminus may compromise how these defined chains interact with binding partners and enzymes. Our method does not require any special equipment and can be conducted with enzymes that are readily available. However, the presence of contaminating unlabeled chains limits the types of experiments that can be performed. The FRET-based assay for monitoring ubiquitin insertion into the Cdc48-UN pore, for example, was conducted under single-turnover conditions with excess motor and cofactor, such that contaminating unlabeled chains had no effect on the measurements. Labeled chains generated with our method are also heterogeneous in length, but the approach could be adapted for studies that require polyubiquitins of defined lengths.

## Troubleshooting

### Problem 1

The cysteine-containing ubiquitin sample is in a buffer with thiol-containing reagents (DTT, beta-Mercaptoethanol, etc.) that can react with maleimide conjugated fluorophores. See before you begin: conjugation of Cy3 to cysteine-ubiquitin constructs (step 23).

### Potential solution

Perform a dialysis or desalting step with a 7K MWCO spin desalting column (Thermo Fisher Scientific, Cat. No. 89882) to exchange for buffer compatible with maleimide labeling.

### Problem 2

Following conjugation, my cysteine-ubiquitin sample did not elute from the size-exclusion chromatography column or eluted in the void peak. See before you begin: conjugation of Cy3 to cysteine-ubiquitin constructs (step 24).

### Potential solution

Attachment of hydrophobic fluorophores to ubiquitin can greatly alter its properties and lead to aggregation or adsorption to filter membranes. We have achieved the best results with sulfonated fluorophores that are more hydrophilic and water soluble. The stability of the fluorophore-conjugated ubiquitin can also depend on the labeling position. Ubiquitin labeled at an N-terminal cysteine may be more well-behaved than ubiquitin labeled at an engineered internal cysteine.

### Problem 3

My fluorophore labeling efficiency is low. See before you begin: conjugation of Cy3 to cysteine-ubiquitin constructs (step 25).

### Potential solution

Ensure that the cysteine-containing ubiquitin sample is in a buffer free of thiol-containing reagents. Oxidation of cysteine during the purification steps prior to conjugation will decrease the labeling efficiency. Include a reducing agent (TCEP, DTT, etc.) throughout the purification to keep cysteines reduced.

### Problem 4

During the first round of chain extension on ^Cy3^Ub-His, a diffuse distribution of chains was formed. See step-by-step method details: Assembly and purification of polyubiquitin chains with a Cy3 fluorophore at the first ubiquitin (step 2).

### Potential solution

Decrease the amount of unlabeled ubiquitin in the reaction. If the concentration of unlabeled ubiquitin is too high, the processive E2-E3 ligase gp78RING-Ube2g2 will form a broad length distribution and distinct bands will not be visible on the gel.

### Problem 5

The major product of the elongation of Ub-His with ^Cy3^Ub is a ubiquitin trimer (Ub-^Cy3^Ub-^Cy3^Ub) or longer chain (Ub-^Cy3^Ub-^Cy3^Ub_n_). See step-by-step method details: Assembly and purification of polyubiquitin chains with a Cy3 fluorophore at the second ubiquitin (step 9).

### Potential solution

As shown in the example gel (Figure 2A), the major ubiquitination product formed can vary depending on the molar ratio of unlabeled to labeled ubiquitin. We recommend running small-scale reactions with different concentrations of unlabeled and labeled species to determine the best conditions for a single addition of ^Cy3^Ub to Ub-His.

### Problem 6

The Cy3 fluorescence emission peak is masked by the Cy5 fluorescence emission peak originating from direct excitation of excess Cy5 fluorophores. See step-by-step method details: FRET-based assay of polyubiquitin engagement by Cdc48-Ufd1/Npl4 (step 17).

### Potential solution

A lower concentration of ^Cy5^Cdc48 could be used in the assay to decrease the size of the Cy5 emission peak, but doing so may compromise the extent of donor quenching if the motor is no longer at concentrations that are saturating for polyubiquitin-chain binding. Another potential solution is to decrease the number of Cy5 fluorophores conjugated to Cdc48 hexamers by performing labeling reactions with lower concentrations of Cy5-maleimide.

## Resource availability

### Lead contact

Further information and requests for resources and reagents should be directed to and will be fulfilled by the lead contact, Andreas Martin (a.martin@berkeley.edu).

### Materials availability

This study did not generate new unique reagents.

## Data Availability

• All data generated or analyzed during this study are included in this manuscript. Original SDS-PAGE images scanned for Cy3 are deposited at Mendeley and are publicly available. Mendeley Data: https://data.mendeley.com/datasets/smzx3wz34w/draft?a=2969dbbb-3bd3-4f4a-a60b-3a0e5d0afba7. • This paper does not report original code. • Any additional information required to reanalyze the data reported in this paper is available from the lead contact upon request.
